# The monocyte-derived cytokine response in whole blood from preterm newborns against sepsis-related bacteria is similar to term newborns and adults

**DOI:** 10.3389/fimmu.2024.1353039

**Published:** 2024-03-18

**Authors:** Jop Jans, Sven C. J. van Dun, Renske Gorissen, Roel F. A. Pieterman, Tess S. Voskamp, Sam Schoenmakers, Hendrik Robert Taal, Wendy W. J. Unger

**Affiliations:** ^1^ Laboratory of Pediatrics, Department of Pediatrics, University Medical Center Rotterdam, Erasmus Medical Center - Sophia, Rotterdam, Netherlands; ^2^ Department of Obstetrics and Gynaecology, Erasmus Medical Center, Rotterdam, Netherlands; ^3^ Department of Neonatal and Paediatric Intensive Care, Division of Neonatology, Erasmus University Medical Center, Rotterdam, Netherlands

**Keywords:** newborn – immunology, sepsis, prematurity, bacteria, innate immunity, cytokines

## Abstract

**Introduction:**

Sepsis is characterized by a dysregulated innate immune response. It is a leading cause of morbidity and mortality in newborns, in particular for newborns that are born premature. Although previous literature indicate that the pro-inflammatory response may be impaired in preterm newborns, serum levels of monocyte-derived cytokines, such as TNF-α and IL-6, vary highly between newborns and can reach adult-like concentrations during sepsis. These contradictory observations and the severe consequences of neonatal sepsis in preterm newborns highlight the need for a better understanding of the pro-inflammatory cytokine response of preterm newborns to improve sepsis-related outcomes.

**Methods and results:**

Using an *in vitro* model with multiple read outs at the transcriptional and protein level, we consistently showed that the monocyte-derived cytokine response induced by sepsis-related bacteria is comparable between preterm newborns, term newborns and adults. We substantiated these findings by employing recombinant Toll-like receptor (TLR) ligands and showed that the activation of specific immune pathways, including the expression of TLRs, is also similar between preterm newborns, term newborns and adults. Importantly, we showed that at birth the production of TNF-α and IL-6 is highly variable between individuals and independent of gestational age.

**Discussion:**

These findings indicate that preterm newborns are equally capable of mounting a pro-inflammatory response against a broad range of bacterial pathogens that is comparable to term newborns and adults. Our results provide a better understanding of the pro-inflammatory response by preterm newborns and could guide the development of interventions that specifically modulate the pro-inflammatory response during sepsis in preterm newborns.

## Introduction

During the neonatal period, sepsis is the most common infectious condition leading to life-threatening consequences with a high degree of morbidity and mortality (1). *Escherichia coli*, *Streptococcus agalactiae* (group B streptococcus, GBS), *Staphylococcus aureus* and *Staphylococcus epidermidis* (coagulase negative staphylococcus, CNS) are the most prevalent bacteria to cause neonatal sepsis (2). These bacteria can induce a dysregulated systemic inflammatory response in newborns that leads to sepsis by activating pathogen-recognition receptors (PRRs), e.g. Toll-like receptors (TLRs), on innate immune cells. After activation of PRRs, innate immune cells produce a variety of cytokines, including the pro-inflammatory cytokines interleukin 6 (IL-6) and tumor necrosis factor alpha (TNF-α). These two cytokines are important pro-inflammatory cytokines in sepsis and high levels of IL-6 and TNF-α are correlated with increased disease severity and mortality during sepsis (3–6). The incidence of neonatal sepsis is higher among preterm newborns, defined as newborns that are born before 37 weeks of gestation, compared to term newborns (7). During neonatal sepsis in preterm newborns, serum cytokine levels of IL-6 and TNF-α can reach up to 10,000 pg/mL and 1,000 pg/mL respectively (3, 8, 9). A high degree of variation in cytokine production between individual preterm newborns, with up to 1,000-fold differences, has been observed during sepsis (3). In addition, these serum cytokine levels during sepsis in preterm newborns are comparable with serum cytokine levels during sepsis in term newborns and adults (4, 8, 10, 11). Together these data indicate that during sepsis (a) newborns can mount a significant pro-inflammatory cytokine response comparable with adults independent of gestational age and (b) major differences in cytokine production between newborns are present. The observed differences in serum cytokine levels between newborns during sepsis may result from external factors such as differences in bacterial load or timing of sample collection after onset of infection. On the other hand, intrinsic factors such as sex or genetic background may also be responsible for the observed differences in cytokine responses. To shed light on the source causing the variation between newborns, *in vitro* assays using whole blood (WB) with set concentrations of stimuli and time points can be useful. Such *in vitro* models allow for assessment of the effect of prematurity by investigating the cytokine response in WB samples from newborns with different gestational ages. Additionally, these models could provide a better understanding of the pro-inflammatory cytokine response of preterm newborns at birth. Previous studies mainly focused on the pro-inflammatory cytokine response of term newborns rather than preterm newborns (12–16). Studies using preterm cord blood are scarce and show conflicting results. Compared to term newborns, the production of IL-6 and TNF-α by preterm newborns has been shown to be either lower, comparable or higher (17–22). Differences in experimental setup between these studies, e.g. differences in cell isolation, time points, stimuli or read outs, may be responsible for the conflicting results. These contradictory observations and the severe consequences of neonatal sepsis in preterm newborns highlight the need for a better understanding of the pro-inflammatory cytokine response of preterm newborns to improve sepsis-related outcomes. In this study, we will provide a detailed analysis of the production of IL-6 and TNF-α by preterm and term newborns at birth in response to different stimuli, such as various sepsis-related bacteria and TLR ligands. In addition, this study employs complementary readouts, such as cytokine gene expression levels in addition to protein levels, to further strengthen our analyses.

## Materials and methods

### Sample processing and stimulation

Human newborn blood was collected directly after birth from the umbilical cord. Newborns from mothers with clinical signs of chorioamnionitis (23) or HIV-positive mothers were excluded. Clinical characteristics such as gestational age, birth weight, sex, mode of delivery, the use of antenatal steroids, the medical indication for cesarean section or prematurity and the presence of early onset neonatal sepsis were recorded. Term WB was defined as blood collected from the umbilical cord of pregnancies with a gestational age ≥ 37 + 0 weeks and preterm neonatal WB was defined as cord blood collected from the umbilical cord of pregnancies with a gestational age < 37 + 0 weeks. Peripheral blood was collected from healthy adults and defined as adult WB. Experimental guidelines of the Regional Committee on Research involving Human Subjects at the Erasmus Medical Center in Rotterdam were observed and the study was approved by the local institutional review board. Written informed consent was obtained from subjects, parents or legal guardians. Blood was collected in lithium heparin tubes (BD Vacutainer) and processed within 2 hours after collection. The number of white blood cells, monocytes, lymphocytes and granulocytes was determined by cell counter (Beckmann Coulter).

### Bacterial culture

The following bacterial strains were used: *E. coli* DH5, *S. aureus* (ATCC 25923), *S. epidermidis* (CNS) (ATCC 12228) and *S. agalactiae* (GBS) (ATCC 27956). Bacterial stocks were plated on blood agar plates (BD™ Trypticase™ Soy Agar II with 5% Sheep Blood) o/n at 37°C and 5% CO_2_. Bacterial suspensions were made with a single colony and OD600 was measured in duplicate with Versamax™ Microplate reader (Molecular Devices). OD600 was set on 0.6 (equivalent to 5 x 10^8^ CFU/mL). Specific dilutions were plated on LB agar or Blood agar and incubated overnight at 37°C. Colonies were counted the next day and suspensions were aliquoted and stored at -80°C.

### Stimulation assay

After collection, blood was subsequently diluted 5-fold with RPMI medium (Gibco) and stimulated with TLR ligands or live bacteria. The following TLR ligands were used: lipopolysaccharide (LPS, TLR4 ligand; Invitrogen), Pam3CysK4 (Pam, TLR2/1 ligand; InvivoGen) and R848 (TLR7/8 ligand; InvivoGen). The concentrations used for all stimuli are depicted in the figure legends. A volume of 180 µl of diluted WB was incubated with 20 µl of stimuli at 37°C and 5% CO_2_ in a 96-well U-bottom culture plate for 5h. For intracellular cytokine detection, 5 µg/mL of Brefeldin A (Invitrogen) was added during the last 2.5h. After stimulation, plates were centrifuged at 500 x *g.* Supernatant was stored at -80°C for ELISA and cells were either stored in RNAlater (Thermo Fisher Scientific) for qPCR or analyzed for intracellular staining and flow cytometry. All conditions were performed in duplicate.

### Enzyme-linked immunosorbent assay

ELISA was performed according to the manufacturer’s protocol. Immuno™ Maxisorp™ plates (NUNC-Thermo Fisher Scientific, 442404) were coated overnight at 4°C with anti-human IL-6 (eBioscience™), anti-human TNF-α antibodies (BD Pharmingen™) or anti-human IL-1β antibodies (R&D Systems). Plates were washed and blocked with phosphate buffered saline (PBS) containing 1% bovine serum albumin (BSA) for 2h at 37°C. Plates were washed and incubated with supernatant or standard dilutions of recombinant human TNF-α (BD Pharmingen™), IL-6 (Thermo Fisher Scientific) or IL-1β (R&D Systems). Biotin Mouse anti-Human TNF-α detection antibody (BD Pharmingen™), Biotin Mouse Anti-Human IL-6 detection antibody (eBioscience™) or Biotin mouse anti-Human IL-1β detection antibody (R&D Systems) was added as secondary antibody. RPMI medium was used as a negative control. Serum from LPS-activated WB was used as a positive control. Plates were washed and incubated with 3,3′,5,5′-tetramethylbenzidine (TMB) after which the reaction was stopped with sulfuric acid (0.8 M H_2_SO_4_). The absorbance was measured at OD450 nm on a SpectraMax^®^ iD3 Reader with SoftMax Pro 7 software. All conditions were performed in duplicate.

### Intracellular cytokine detection

After stimulation, samples were centrifuged at 500 x *g* for 3 min at 4°C. Cells were resuspended in 200 µl PBS (Gibco) supplemented with 2% FCS (Bodinco) and 2 mM EDTA (hereafter referred to as FACSbuffer) and washed. Cell pellets were incubated for 5 minutes with RBC lysis buffer (0.15 M NH_4_Cl, 10 mM KHCO_3_, 0.1 mM EDTA). If necessary, RBC lysis was repeated. After washing with PBS, cells were incubated for 30 minutes with Live/Dead stain (Invitrogen LIVE/DEAD^®^ Fixable Aqua Dead Cell Stain Kit) at 4°C. Cells were washed and incubated for 30 min at 4°C with the following fluorescent-labeled antibodies (Biolegend): anti-CD16 PE-Cy7 (clone 3G8), anti-CD14 APC-Cy7 (clone MΦP9), anti-HLA-DR PerCp-Cy5.5 (clone G46.6) from BD Pharmingen and anti-CD3 FITC (clone UCHT1), anti-CD19 FITC (clone HIB19), anti-CD56 FITC (clone HCD56) and anti-CD66b Pacific Blue (clone G10F5). Samples were washed and incubated with Fix/Perm solution (BD Cytofix/Cytoperm Fixation/Permeabilisation kit) at 4°C for 20 min. Thereafter, samples were incubated with α-IL-6 PE (clone MQ2-13A5) and α-TNF-α APC (clone Mab11) (Invitrogen) diluted in Perm buffer for 30 min at 4°C. Cells were washed and events were acquired at a BD FACSCanto™ II Flow Cytometer. All conditions were performed in duplicate. Analysis was performed using FlowJo version X (BD Biosciences).

### Quantitative polymerase chain reaction

Expression levels of selected genes were measured by quantitative polymerase chain reaction (qPCR) analysis. RNA was extracted using the Nucleospin^®^ RNA extraction kit (Macherey-nagel) according to the manufacturer’s instructions and eluted in 40 µl RNase free water. Total RNA was transcribed using the sensiFAST™ cDNA synthesis kit (Meridan biosience). The mRNA expression levels of *il6* and *tnf* were analysed by real-time PCR using SensiMix™ SYBR^®^ Hi-ROX Kit (Meridan bioscience). The mRNA levels of *tlr1, tlr2, tlr4, tlr7, tlr8* were analyzed using probe-based multiplex qPCR and Taqman universal PCR mastermix (Applied biosystems). Amplification conditions were according to manufacturer’s instructions, 95°C for 10 minutes polymerase activation, following 40 cycles of 95°C for 15 seconds and 60°C for 1 minute. Amplifications were performed in duplicate. Data are normalized to housekeeping gene ribosomal protein lateral stalk subunit P0 (*rplp0*) expression and depicted as log-transformed 2^Δ-Ct. Primer and probe sequences are shown in **Supplementary Tables 1A, B**.

### Statistical analyses

Comparison of cytokine production, mRNA expression levels and white blood cell counts between preterm neonatal WB, term neonatal WB and adult WB employed one-way ANOVA with Bonferroni’s Multiple Comparison Test. Inter-individual variation was determined by calculating the coefficiency of variation (CV). CV is defined as standard deviation divided by the median (24). Tests were considered statistically significant if *P*<0.05. Spearman’s rank correlation coefficient was used as a non-parametric measure of rank correlation between the production of TNF-α and IL-6. All statistical analyses were done with GraphPad Prism.

## Results

### The production of TNF-α and IL-6 by term neonatal and adult WB shows a high degree of inter-individual variation

**Figure 1:**
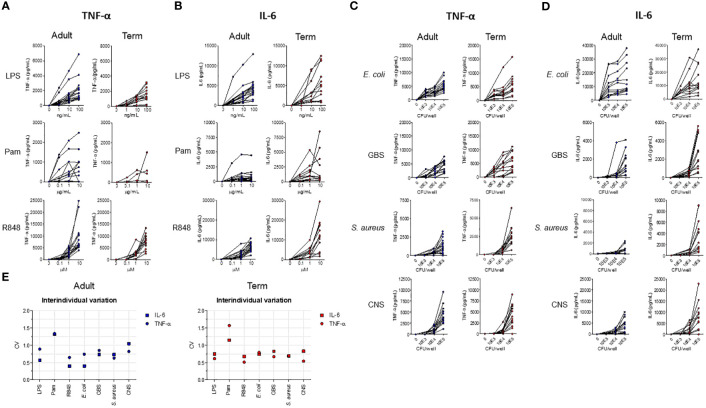
The production of TNF-α and IL-6 by term neonatal and adult WB shows a high degree of inter-individual variation. WB from term cord blood (term, N=11-17) and healthy adults (adult, N=11-18) was incubated with **(A, B)** LPS, Pam3Cys4K or R848 or with **(C, D)** *E. coli*, *S. agalactiae* (GBS), *S. aureus* or *S. epidermidis* (CNS) bacteria for 5h and the production of TNF-α and IL-6 was measured. **(E)** The inter-individual variation in production of TNF-α and IL-6 between individuals was determined for WB from term cord blood and healthy adults (N=11-18) for LPS (100 ng/mL), Pam3Cys4K (10 μg/mL) or R848 (10 μM) or bacteria (10^5^ CFU/well). Cytokine concentrations are shown in pg/mL. CFU, colony-forming units.

Serum levels of TNF-α and IL-6 vary highly between newborns during sepsis (3, 9, 11). We determined whether the variation in cytokine production between individuals can also be detected in an *in vitro* model with set concentrations of stimuli and time points. An incubation period of 5 hours was chosen, because the production of TNF-α and IL-6 peaked at 5 hours and possible interference of dying neutrophils was absent or minimal (data not shown). A concentration-dependent production of TNF-α and IL-6 was observed after exposure of term neonatal or adult WB to different TLR ligands and sepsis-related bacteria (**Figures 1A–D**). The median levels and range of TNF-α and IL-6 per stimulus for adult and term WB are shown in **Supplementary Tables 2A, B**. To determine the degree of variation in *in vitro* cytokine production, the coefficient of variation (CV) was calculated (24). A CV > 0.3 is considered a high degree of inter-individual variation. The CV of TNF-α and IL-6 production showed a high degree of inter-individual variation (range 0.4 – 1.7) for adult and term neonatal WB (**Figure 1E**). Stimulation with Pam3Cys4K resulted in the highest CV of TNF-α and IL-6 production.

### Preterm neonatal WB produces lower levels of IL-6 and similar levels of TNF-α compared to term neonatal WB

**Figure 2:**
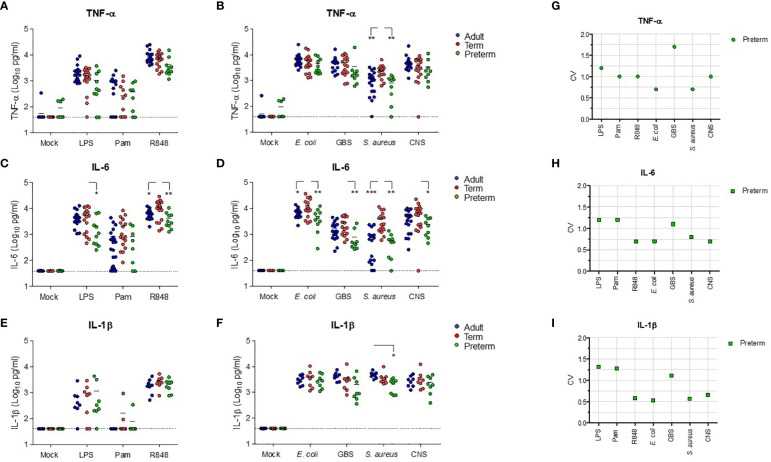
Preterm neonatal WB produces lower levels of IL-6 and similar levels of TNF-α compared to term neonatal WB. Whole blood from preterm cord blood (preterm, N=8-10), term cord blood (term, N=17) and healthy adults (adult, N=17-21) was incubated with TLR ligands LPS (100 ng/mL), Pam3Cys4K (10 μg/mL) or R848 (10 μM) or with *E. coli*, *S. agalactiae* (GBS), *S. aureus* or *S. epidermidis* (CNS) bacteria (10^5^ CFU/well) for 5h and the production of **(A, B)** TNF-α, **(C, D)** IL-6 and **(E, F)** IL-1β was measured. **(G-I)** The inter-individual variation in production of **(G)** TNF-α, **(H)** IL-6 and **(I)** IL-1β between individuals was determined for preterm cord blood (N=8-10). Cytokine concentrations are shown in log-transformed pg/mL. The dotted line indicates the limit of detection. CV = coefficiency of variation. * = P<0.05. ** = P<0.01. *** = P<0.001.

**Figure 3:**
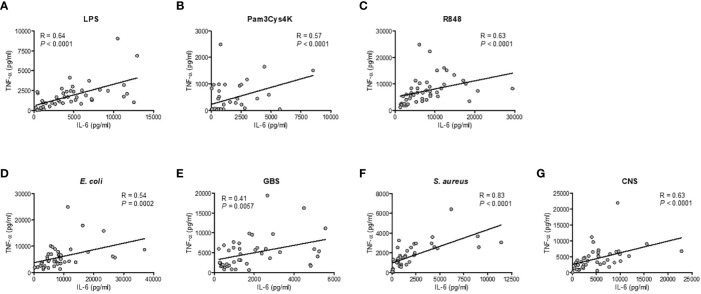
Correlation between the production of TNF-α and IL-6 within individuals. The production of TNF-α and IL-6 for each sample was compared in samples from preterm cord blood, term cord blood and healthy adults to determine the correlation between the production of TNF-α and IL-6 after incubation with **(A–C)** TLR ligands LPS, Pam3Cys4K or R848 and **(D–G)** *E. coli, S. agalactiae* (GBS), *S. aureus* or *S. epidermidis *(CNS) within each individual (N=43-47). Linear regression was performed and Spearman’s test was used to assess correlation.

To evaluate the effect of prematurity on the production of TNF-α and IL-6, we exposed preterm neonatal, term neonatal and adult WB to TLR ligands and sepsis-related bacteria. Clinical characteristics of preterm and term neonatal WB samples are shown in **Table 1**. Preterm neonatal WB produced equal amounts of TNF-α and IL-6 as adult WB, irrespective of the stimulus used (**Figures 2A–D**). Preterm neonatal WB also produced equal amounts of TNF-α as term neonatal WB for all stimuli, except for *S. aureus* (**Figures 2A, B**)*. S. aureus* induced a lower production of TNF-α by preterm neonatal WB (1,009 pg/mL; range 39-1,597 pg/mL) compared to term neonatal WB (2,534 pg/mL; range 625-6,409 pg/mL). Preterm neonatal WB produced lower amounts of IL-6 compared to term neonatal WB for all stimuli, except for Pam3Cys4K. Pam3Cys4K induced equal amounts of IL-6 by preterm and term neonatal WB (**Figures 2C, D**). The median levels and range of TNF-α and IL-6 per stimulus for preterm neonatal, term neonatal and adult WB are shown in **Supplementary Tables 3A, B**. Besides TNF-α and IL-6, also IL-1β drives protective immunity during bacterial infections. We observed that the production of IL-1β by preterm neonatal WB was similar to term neonatal WB and adult WB (**Figures 2E, F**). A high degree of inter-individual variation in the production of TNF-α, IL-6 and IL-1β by preterm neonatal WB was observed (CV range 0.6-1.7) (**Figures 2G–I**). However, in contrast to TNF-α and IL-6, IL-1β serum levels are extremely low (<50 pg/mL) during neonatal sepsis (25). Therefore, in the remainder of the study we focused on TNF-α and IL-6. The signaling pathways that lead to production of TNF-α and IL-6 share multiple molecules, such as adaptors and kinases (26). We therefore investigated the correlation between TNF-α and IL-6 within the same individual. A linear regression analysis showed a correlation between the production of TNF-α and IL-6 within individuals, which was found for all tested stimuli (**Figures 3A–G**). A summary of the inter-individual variation of TNF-α and IL-6 production by preterm neonatal, term neonatal and adult WB is depicted in **Supplementary Table 4**.

**Table 1:** Sample characteristics.

	Term neonatal WB (N=19)	Preterm neonatal WB (N=10)
**Gestational age (range in weeks)**	38.6 (37.3 – 39.6)	31 (26.4 – 35.4)
**Birth weight (range in grams)**	3292 (2420 – 4130)	1506 (750 -2755)
**Sex**	68% female, 32 % male	30% female, 70% male
**Cesarean section (%)**	19/19 (100%)	7/10 (70%)
**Antenatal steroids (%)**	0/19 (0%)	8/10 (80%)
Medical indication for cesarean section/prematurity (%)		
History of cesarean section	14/19 (74%)	0/10 (0%)
Breach	3/19 (16%)	0/10 (0%)
Failure to progress labor	1/19 (5%)	0/10 (0%)
Pre-eclampsia	0/19 (0%)	3/10 (30%)
Other**^1^**	1/19 (5%)	1/10 (10%)
Unknown	0/19 (0%)	6/10 (60%)
**Early onset sepsis (%)^2^**	0/19 (0%)	0/10 (0%)

^1^Maternal tethered cord (N=1), cervical insufficiency (N=1).

^2^Defined as a positive blood culture or clinical symptoms of infection and CRP >20 with negative blood culture within 72h after birth.

### Reduced production of IL-6 by preterm neonatal WB is not due to altered transcriptional levels of *tlr* or *il6*

**Figure 4:**
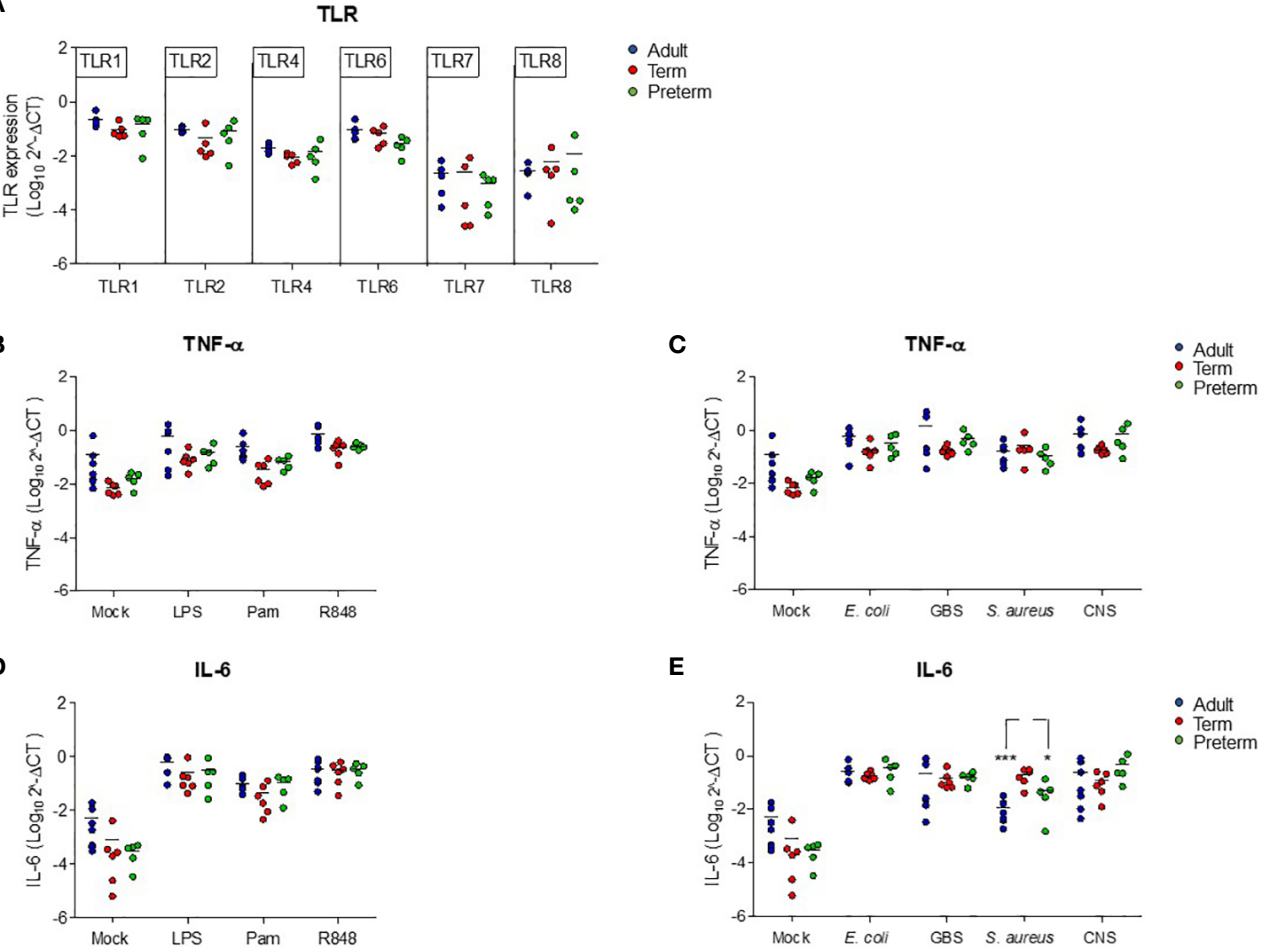
Reduced production of IL-6 in preterm neonatal WB is not due to altered transcriptional levels of *tlr* or *il6.* **(A)** The expression levels of *tlr1, tlr2, tlr4, tlr6, tlr7* and *tlr8* were determined in WB from preterm cord blood (preterm, N=5), term cord blood (term, N=5) and healthy adults (adult, N=6). **(B–E)** The expression levels of **(B, C)** *tnf* and **(D, E)** *il6* were determined in WB from preterm cord blood (preterm, N=5), term cord blood (term, N=6) and healthy adults (adult, N=6-7) unstimulated (mock) or after stimulation with LPS (100 ng/mL), Pam3Cys4K (10 μg/mL) or R848 (10 μM) or with *E coli*, *S. agalactiae* (GBS), *S. aureus* or *S. epidermidis* (CNS) bacteria (10^5^ CFU/well) for 5h. Expression levels are shown as log-transformed 2^ΔCt. * = P<0.05. *** = P<0.001.

To investigate the mechanisms behind the low production of IL-6 by preterm neonatal WB, we first determined the expression of different TLRs that are involved in the production of TNF-α and IL-6 after exposure to TLR ligands and bacteria. No differences in expression levels of *tlr1, tlr2, tlr4, tlr6, tlr7* and *trl8* were observed between preterm neonatal WB, term neonatal WB and adult WB (**Figure 4A**). The induction of *tnf* at the transcriptional level after exposure to all stimuli showed similar results as the production of TNF-α at the protein level. Equal amounts of *tnf* mRNA were induced in preterm neonatal WB, term neonatal WB and adult WB (**Figures 4B, C**). We next investigated whether a defective induction of *il6* at the transcriptional level could explain the low production of IL-6 at the protein level by preterm neonatal WB. Measurement of *il6* mRNA levels showed that the amount of *il6* at the transcriptional level in preterm neonatal WB was similar to term neonatal WB and adult WB, irrespective of the stimulus (**Figures 4D, E**). The only exception was the expression of *il6* mRNA levels after exposure to *S. aureus*, which were lower in preterm neonatal WB compared to term neonatal WB (**Figure 4E**).

### Cell-corrected production of TNF-α and IL-6 by preterm neonatal WB is comparable to term neonatal and adult WB and has a high degree of inter-individual variation

**Figure 5:**
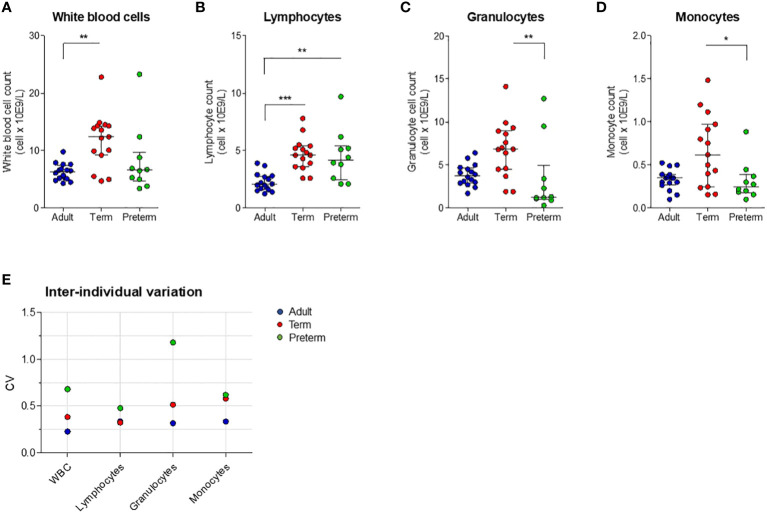
White blood cell count and inter-individual variation between preterm neonatal WB, term neonatal WB and adult WB. **(A–D)** White blood cell counts, including monocyte count, granulocyte count and lymphocyte count, are depicted for preterm neonatal WB (preterm, N=10), term neonatal WB (term, N=15) and adult WB (adult, N=16). **(E)** The inter-individual variation in cell count for preterm neonatal WB, term neonatal WB and adult WB. Cell counts are shown as median ± interquartile range. CV = coefficiency of variation. WBC = white blood cells. * = P<0.05. ** = P<0.01. *** = P<0.001.

**Figure 6:**
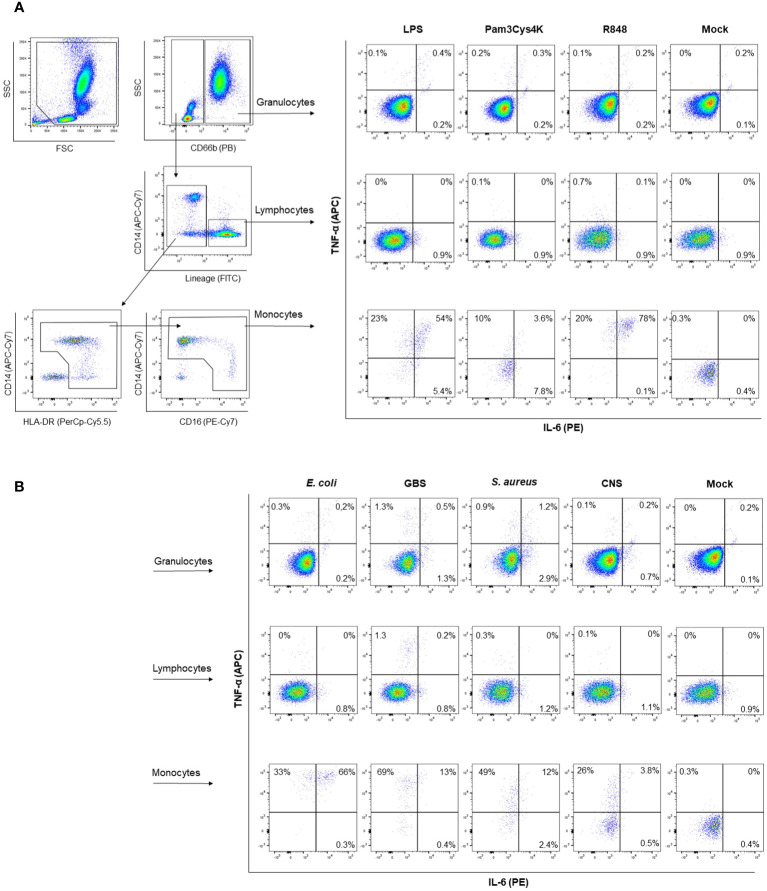
Representative gating strategy for intracellular cytokine staining. A representative gating strategy is depicted to determine which cells produce TNF-α and IL-6. Granulocytes are identified as CD66b^+^ cells. Lymphocytes are identified as lineage positive, being either CD3^+^, CD19^+^ or CD56^+^. Monocytes are identified as CD66b^-^, lineage negative, HLA-DR^+^. Intracellular staining for TNF-α and IL-6 is shown with and without (mock) stimulation with **(A)** TLR ligands or **(B)** sepsis-related bacteria.

**Figure 7:**
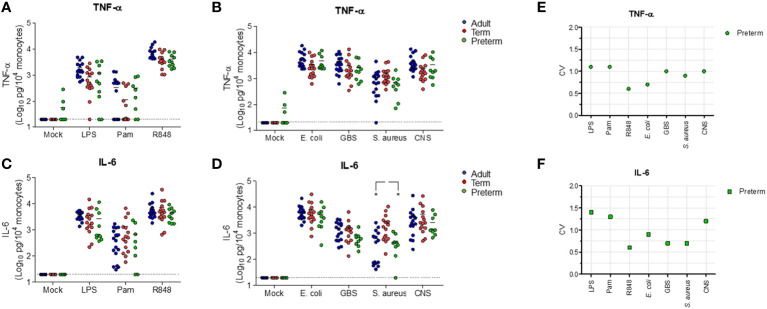
Cell-corrected production of TNF-α and IL-6 by preterm neonatal WB is comparable to term neonatal and adult WB and has a high degree of inter-individual variation. Whole blood from preterm cord blood (preterm, N=9-10), term cord blood (term, N=14-15) and healthy adults (adult, N=16) was incubated with TLR ligands LPS (100 ng/mL), Pam3Cys4K (10 μg/mL) or R848 (10 μM) or with *E. coli*, *S. agalactiae* (GBS), *S. aureus* or *S. epidermidis* (CNS) bacteria (10^5^ CFU/well) for 5h and the monocyte-corrected production of **(A, B)** TNF-α and **(C, D)** IL-6 was measured. Monocyte-corrected production of cytokines was determined as follows: cytokine production/the amount of monocytes per well*10,000. **(E, F)** The inter-individual variation in production of **(E)** TNF-α and **(F)** IL-6 was determined for preterm cord blood (N=9-10). Cell-corrected cytokine concentrations are shown in log-transformed pg/mL per 10^4^ monocytes. The dotted line indicates the limit of detection CV, coefficiency of variation. * = P<0.05.

Having established that transcriptional levels of *tlr, tnf* and *il6* are not different between preterm and term neonatal WB, we hypothesized that the discrepancy between protein and mRNA levels of IL-6 may result from differences in white blood cell (WBC) numbers between individuals. Evaluation of the number of WBC shows a high number in term neonatal WB compared to adult WB (**Figure 5A**). Zooming in on the WBC composition revealed that the lymphocyte count in preterm neonatal and term neonatal WB was higher than the lymphocyte count in adult WB. The granulocyte and monocyte counts in preterm neonatal WB were lower compared to term neonatal WB (**Figures 5B–D**). For all cell types, a high degree of variation in cell count was observed for preterm neonatal WB compared to term neonatal and adult WB (**Figure 5E**). Intracellular cytokine staining was used to assess which cells produced TNF-α and IL-6 and to determine which cells could be used to determine the cell-corrected production of TNF-α and IL-6. After exposure of WB to TLR ligands or bacteria, TNF-α and IL-6 were exclusively detected in monocytes, and not in lymphocytes and granulocytes (**Figures 6A, B**). After correcting for the monocyte count, the amounts of TNF-α and IL-6 produced by preterm neonatal WB was comparable with term neonatal WB and adult WB for all stimuli, expect for *S. aureus* (**Figures 7A–D**). *S. aureus* induced a lower production of IL-6 in preterm neonatal WB (620 pg/mL; range 202 – 1,752 pg/mL) compared to term neonatal WB (1,406 pg/mL; range 135-6,537 pg/mL). The median levels and range of monocyte-corrected production of TNF-α and IL-6 per stimulus are shown in **Supplementary Tables 5A, B**. After correction for monocyte count, the CV of TNF-α and IL-6 production by preterm neonatal WB still showed a high degree of inter-individual variation ranging from 0.6-1.4 (**Figures 7E, F**, **Supplementary Table 6**).

## Discussion

This is the first study to show that the *in vitro* production of TNF-α and IL-6 by preterm neonatal WB at birth is comparable to term neonatal WB and adult WB after exposure to a broad range of stimuli that are relevant during neonatal sepsis. In addition, we have shown that a high degree of inter-individual variation in the production of TNF-α and IL-6 by preterm neonatal WB is present at birth.

Our initial experiments indicated that preterm neonatal and adult WB produced low levels of IL-6 compared to term neonatal WB after exposure to TLR ligands and live bacteria. However, after correction for monocyte counts, our study shows that the intrinsic capacity of preterm neonatal WB to produce monocyte-derived TNF-α and IL-6 is comparable to term neonatal and adult WB. A correlation between the production of TNF-α and IL-6 within each sample was observed in this study. In addition, we showed that the production of IL-1β was also similar between preterm neonatal WB and term neonatal WB. The high production of multiple pro-inflammatory cytokines within the same individual could explain the cytokine storm that is observed in neonates that develop sepsis during infection. We used different read outs, such as TLR expression levels, transcriptional levels of *tnf* and *il6* and monocyte-corrected production of TNF-α and IL-6 to clearly show that the intrinsic capacity of preterm newborns to produce TNF-α and IL-6 is comparable to term newborns and adults. Our study highlights the importance of using different readouts and correction for cell types when comparing WB between different populations. Contrary to our results, previous studies have observed a reduced production of cytokines by preterm neonatal WB (21, 22). However, such studies did not consider differences in white blood cell counts between individuals nor measured the transcriptional levels of cytokines. Also differences in study design compared to our study could explain the differences in result. Studies involving stimulation of peripheral blood mononuclear cells (PBMCs) or cord blood mononuclear cells (CBMCs) also showed a reduced production of cytokine by preterm newborns compared to term newborns (17, 19, 27). These studies employed a plasma-free model with isolated mononuclear cells in contrast to our WB model with all plasma components present. Different plasma components, e.g. antibodies, complement or adenosine, can influence the *in vitro* production of cytokines (28–30) which may explain the differences in results between these previous studies and our results. In addition, interaction between monocytes and neutrophils is not present in a model with isolated mononuclear cells, but this interaction can play a role in the production of TNF-α and IL-6 by monocytes (31). Therefore, we decided against isolating cells prior to our stimulation assay and to mimic the inflammatory response during sepsis as physiological as possible. Our *in vitro* data is supported by clinical data that show comparable serum levels of TNF-α and IL-6 during sepsis between preterm newborns, term newborns and adults (4, 8, 10, 11).

*S. aureus* was the only stimulus that consistently induced a lower production of IL-6 in preterm neonatal WB compared to term neonatal WB throughout all read outs, being the transcriptional level, the protein level and the production of IL-6 after correction for monocyte counts. Levels of maternal antibodies could potentially play a role in the lower production of IL-6 in preterm neonatal WB, because preterm newborns in general have lower maternal antibody levels compared to term newborns. While opsonization of *S. aureus* by antibodies can amplify the production of IL-6 by dendritic cells via synergy with TLR stimulation, this mechanism is not effective in monocytes (30). Considering that monocytes are the main producers of IL-6 in our model, possible differences in maternal antibody levels between preterm and term newborns most likely do not explain the lower production of IL-6 by preterm neonatal WB after exposure to *S. aureus*. Although *S. aureus* induces many pathways that affect both TNF-α and IL-6 production, α-toxins are important toxins produced by *S. aureus* and specifically induce IL-6 in humans and mice, whereas TNF-α is not induced by α-toxins (32, 33). Therefore, the low production of IL-6 by preterm neonatal WB in our study could be explained by a low expression of innate immune receptors, that are known to bind α-toxins and induce IL-6, e.g. Nod-like receptors (NLRs) (34). Our study raises the clinical question whether the high incidence of *S. aureus* infections in hospitalized preterm newborns (35) is due to the low production of IL-6 by preterm newborns. Strunk et al. showed that a low *in vitro* production of IL-6 to *S. epidermidis* is associated with an increased risk of *S. epidermidis* infections (36). A clinical study that correlates the *in vitro* response against *S. aureus* with the occurrence of *S. aureus* infections in preterm newborns could give novel insights into the protective role of IL-6 against *S. aureus* infections in preterm newborns.

Finally, we observed a high degree of inter-individual variation in cytokine production by preterm neonatal WB at birth. These major differences in the production of TNF-α and IL-6 between individuals are also observed in serum samples collected during neonatal sepsis (3). By using cord blood, this study shows that the inter-individual variation in cytokine production is not only present during sepsis but already *in vitro* at birth independent of gestational age. Because the variation in cytokine production is already present at birth, genetic or epigenetic factors could contribute to the variation between individuals with regards to cytokine production and disease severity. Indeed, previous literature shows that genetic polymorphisms in innate immune signaling factors, such as IL-6, TLR2 and IL-1β may explain the variation in disease severity during bacterial infections in preterm and term newborns (37, 38). In addition, epigenetic changes play a role during sepsis by modulating the production of TNF-α and IL-6 (39). Histone deacetylases (HDACs) are one example of epigenetic regulators that control chromatin structure and gene expression. The role of epigenetics in the context of bacterial infections is supported by previous literature showing that HDACs are essential for LPS-induced inflammation by monocytic cells (40) and inhibition of HDACs attenuates the harmful pro-inflammatory response by LPS *in vivo* (41).

Our study has several limitations. The percentage of antenatal steroid administration prior to the sample collection is higher for preterm neonatal WB compared to term neonatal WB. Although steroids in general can have an immunosuppressive effect, previous literature has shown that antenatal administration does not affect the *in vitro* cytokine response of cord blood (42). The analysis of mRNA levels in WB limits the ability to determine which cell type in our assay contains *tlr*, *tnf* and *il6* mRNA. Nevertheless, despite this limitation, intracellular cytokine staining has shown that monocytes produce TNF-α and IL-6 in our assay. Therefore, we think that a combined approach by utilizing intracellular cytokine staining, assessing mRNA expression levels and adjusting for monocyte counts in WB is essential for an accurate evaluation of the intrinsic capacity of preterm newborns to mount a pro-inflammatory cytokine response.

In summary, we provide an extensive description of the monocyte-derived pro-inflammatory cytokine responses by preterm newborns and show that TLR-mediated production of TNF-α and IL-6 in the context of bacterial infections is comparable between preterm neonatal, term neonatal and adult WB. The use of different stimuli, including TLR ligands and live bacteria, has provided valuable information on multiple signaling pathways of the innate immune response in preterm newborns. Our findings suggest that a high degree of inter-individual cytokine response is already present at birth, which may indicate a window of opportunity directly after birth to identify individuals with an intrinsically high pro-inflammatory cytokine response. Future studies to decipher the mechanisms that can lead to an exuberant production of TNF-α and IL-6 and to better understand the detrimental inflammatory response in those newborns that progress to sepsis during a bacterial infection are essential. A better understanding of the innate immune response by preterm newborns could guide optimal interventions such as immunomodulation or epigenetic regulators to better protect newborns that are at risk for sepsis.

## Data availability statement

The raw data supporting the conclusions of this article will be made available by the authors, without undue reservation.

## Ethics statement

The studies involving humans were approved by the Regional Committee on Research involving Human Subjects Erasmus Medical Center Rotterdam. The studies were conducted in accordance with the local legislation and institutional requirements. Written informed consent for participation in this study was provided by the participants’ legal guardians/next of kin. Written informed consent was obtained from the individual(s), and minor(s)’ legal guardian/next of kin, for the publication of any potentially identifiable images or data included in this article.

## Author contributions

JJ: Conceptualization, Data curation, Formal analysis, Funding acquisition, Investigation, Methodology, Project administration, Supervision, Visualization, Writing – original draft, Writing – review & editing. SD: Writing – review & editing, Data curation, Formal analysis, Methodology. RG: Data curation, Formal analysis, Methodology, Writing – review & editing. RP: Methodology, Writing – review & editing. TV: Writing – review & editing, Data curation, Formal analysis, Methodology. SS: Writing – review & editing. HT: Writing – review & editing, Investigation. WU: Conceptualization, Funding acquisition, Methodology, Project administration, Supervision, Writing – original draft, Writing – review & editing.

## Supplementary material

Supplementary Table 1 Primer/probe sequences.

Supplementary Table 2 The median levels and range of **(A)** TNF-α and **(B)** IL-6 in pg/mL per stimulus for adult and term neonatal WB.

Supplementary Table 3 The median levels and range of **(A)** TNF-α and **(B)** IL-6 in pg/mL per stimulus for adult WB, term neonatal WB and preterm neonatal WB.

Supplementary Table 4 The coefficiency of variation (CV) median levels and range of TNF-α and IL-6 in pg/mL per stimulius for adult WB, term neonatal WB and preterm neonatal WB.

Supplementary Table 5 The median levels and range of **(A)** TNF-α and **(B)** IL-6 in pg/mL after correction for monocyte count per stimulus for adult WB, term neonatal WB and preterm neonatal WB.

Supplementary Table 6 The coefficiency of variation (CV) median levels and range of TNF-α and IL-6 in pg/mL after correction for monocyte count per stimulus for adult WB, term neonatal WB and preterm neonatal WB.
