# Supplementary figures and images for: The monocyte-derived cytokine response in whole blood from preterm newborns against sepsis-related bacteria is similar to term newborns and adults
Source: Front Immunol. 2024 Mar 18;15:1353039. doi: 10.3389/fimmu.2024.1353039 (PMC10982322; doi:10.3389/fimmu.2024.1353039)

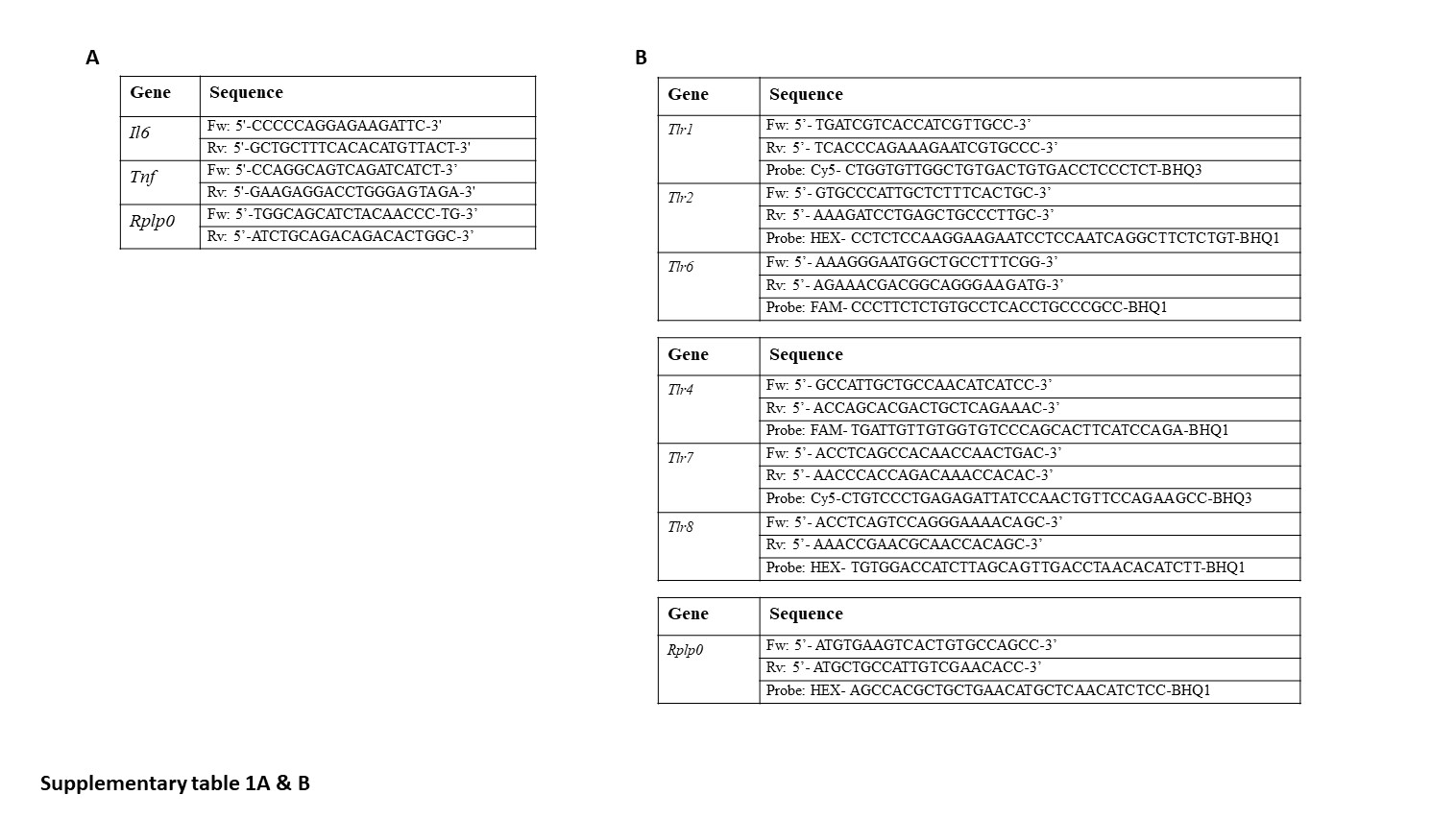

Supplement: Supplementary Table 1 — Primer/probe sequences. [file Image_1.jpeg]

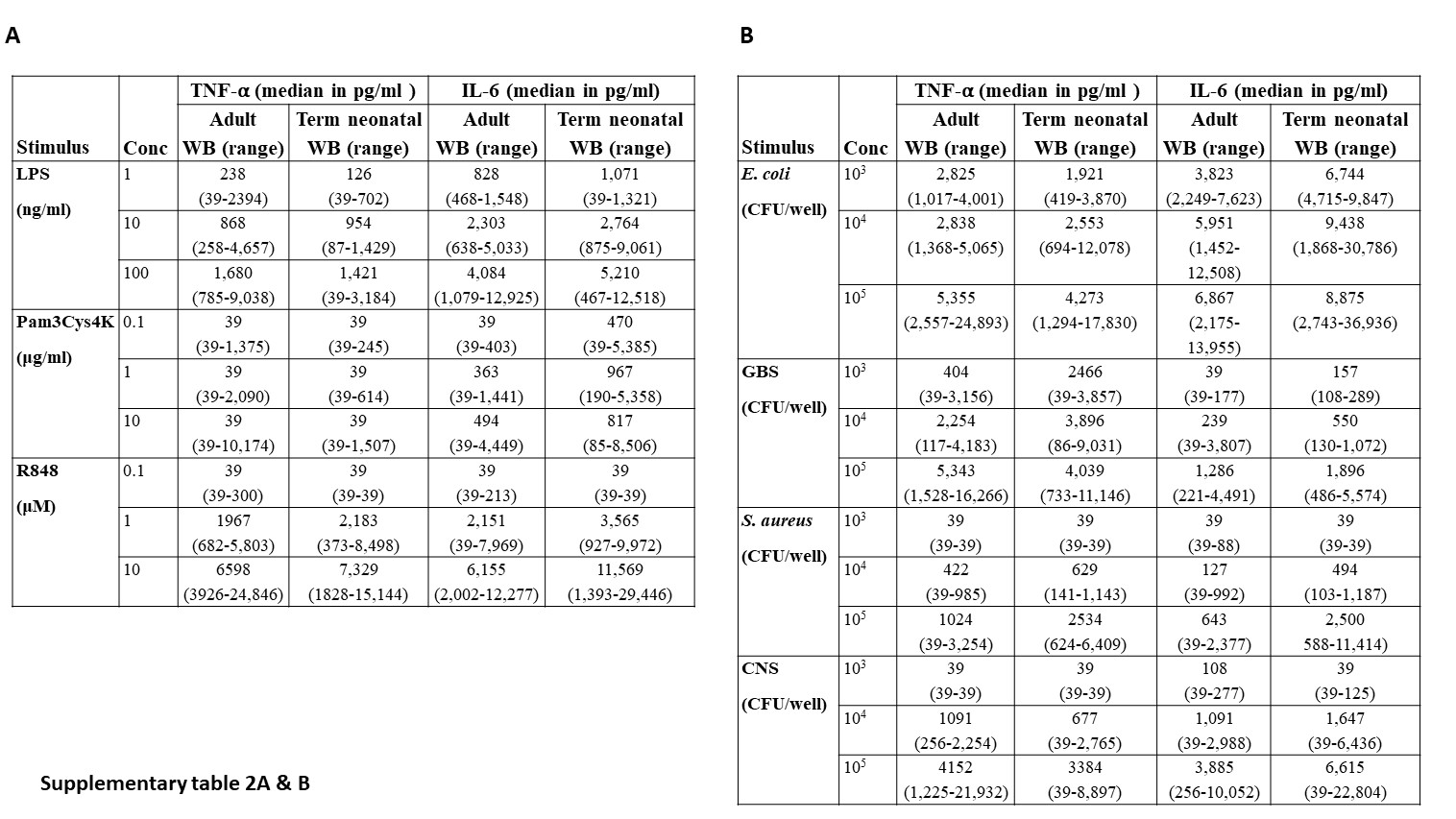

Supplement: Supplementary Table 2 — The median levels and range of (A) TNF-α and (B) IL-6 in pg/mL per stimulus for adult and term neonatal WB. [file Image_2.jpeg]

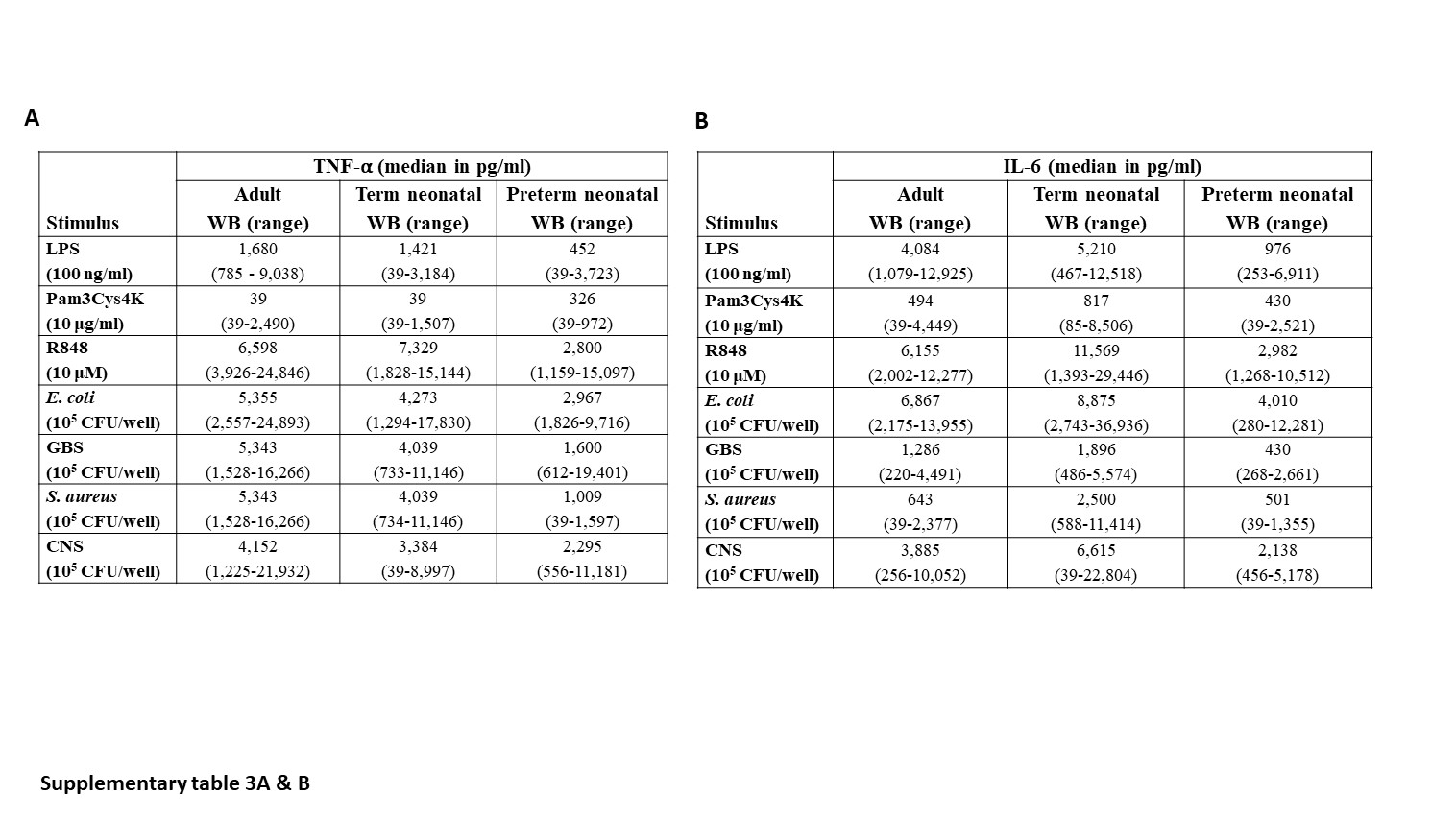

Supplement: Supplementary Table 3 — The median levels and range of (A) TNF-α and (B) IL-6 in pg/mL per stimulus for adult WB, term neonatal WB and preterm neonatal WB. [file Image_3.jpeg]

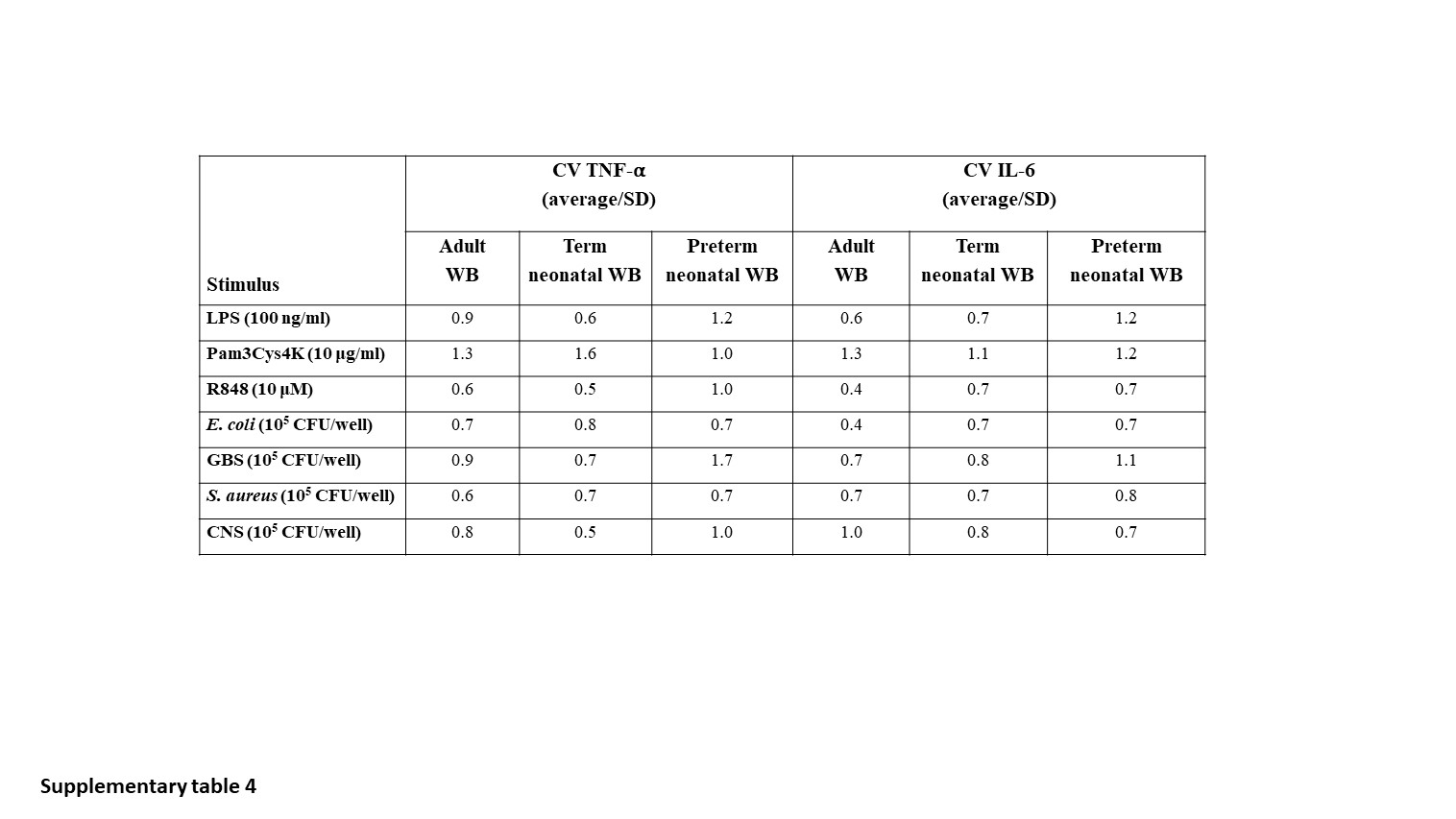

Supplement: Supplementary Table 4 — The coefficiency of variation (CV) median levels and range of TNF-α and IL-6 in pg/mL per stimulius for adult WB, term neonatal WB and preterm neonatal WB. [file Image_4.jpeg]

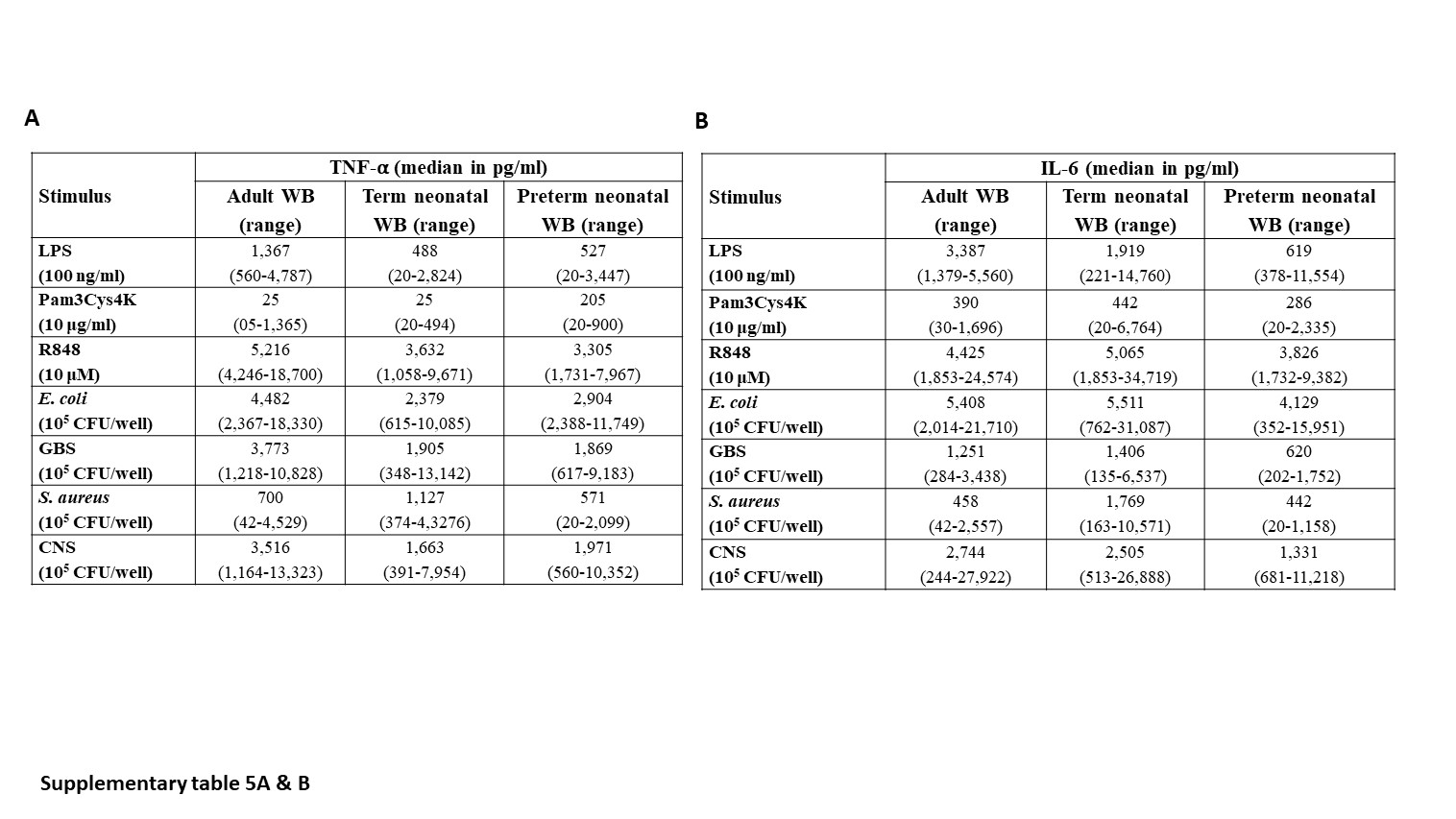

Supplement: Supplementary Table 5 — The median levels and range of (A) TNF-α and (B) IL-6 in pg/mL after correction for monocyte count per stimulus for adult WB, term neonatal WB and preterm neonatal WB. [file Image_5.jpeg]

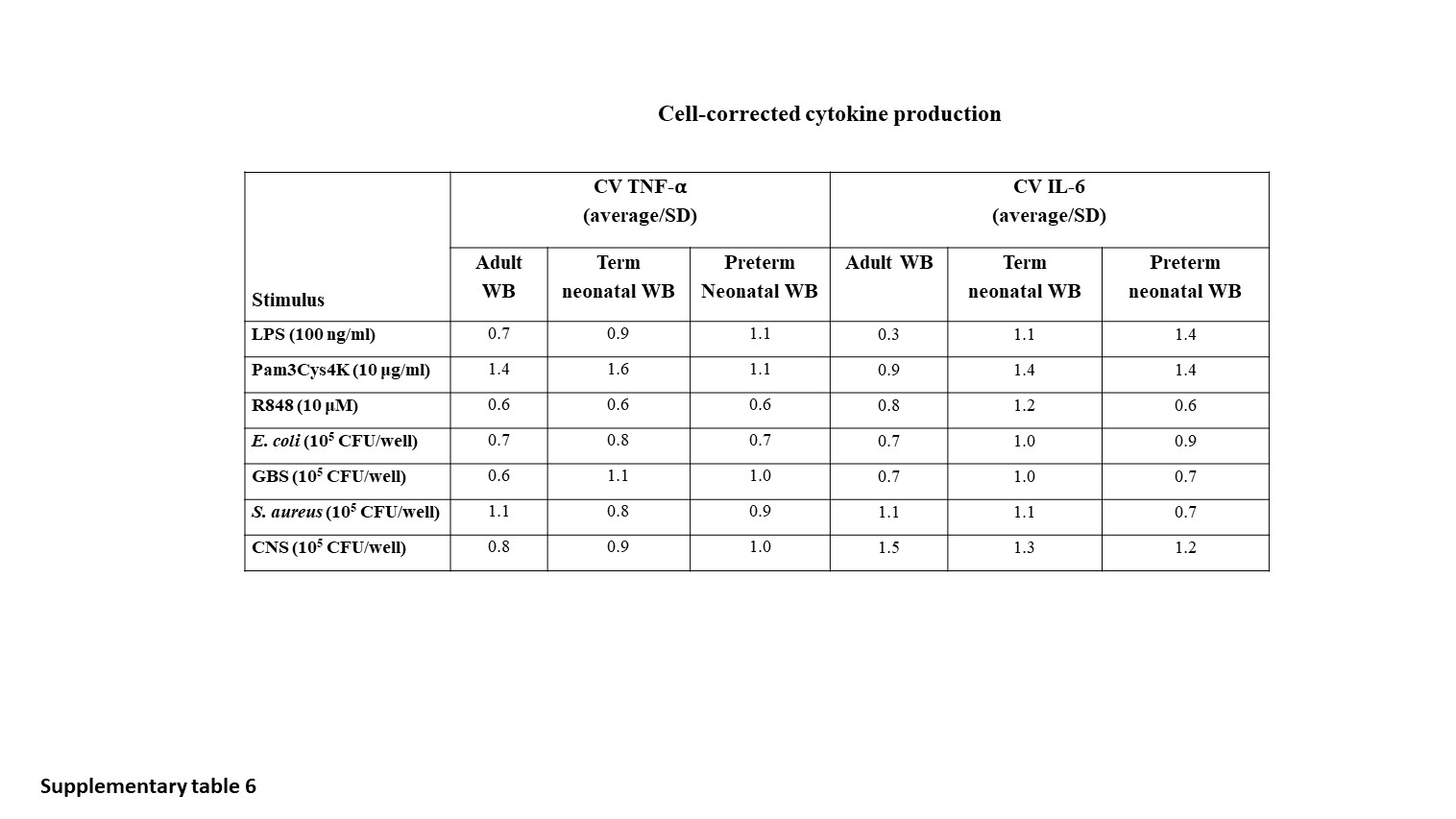

Supplement: Supplementary Table 6 — The coefficiency of variation (CV) median levels and range of TNF-α and IL-6 in pg/mL after correction for monocyte count per stimulus for adult WB, term neonatal WB and preterm neonatal WB. [file Image_6.jpeg]
